# Unsuspected and extensive transmission of a drug-susceptible *Mycobacterium tuberculosis *strain

**DOI:** 10.1186/1471-2466-9-3

**Published:** 2009-01-14

**Authors:** Ana Isabel López-Calleja, Patricia Gavín, Ma Antonia Lezcano, Ma Asunción Vitoria, Ma José Iglesias, Joaquín Guimbao, Ma Ángeles Lázaro, Nalin Rastogi, Ma José Revillo, Carlos Martín, Sofia Samper

**Affiliations:** 1 Servicio de Microbiología, Hospital Universitario Miguel Servet, Paseo Isabel la Católica, Zaragoza, Spain; 2 Instituto Aragonés de Ciencias de la Salud Avda Gómez Laguna, Zaragoza, Spain; 3 Centro de Investigación Biomédica en Red de Enfermedades Respiratorias (CibeRes) Fundación Caubet-Cimera, Recinto Hospital Joan March, Carretera Soller Km 12, 07110 Bunyola, Mallorca, Spain; 4 Servicio de Microbiología, Hospital Clínico Universitario Lozano Blesa Avda Gómez Laguna, Zaragoza, Spain; 5 Departamento de Microbiología, Medicina Preventiva y Salud Pública, Universidad de Zaragoza. c/Domingo Miral, Zaragoza, Spain; 6 Sección de Vigilancia Epidemiológica, Subdirección Provincial de Salud Pública, C/Ramón y Cajal, Zaragoza, Spain; 7 Tuberculosis and Mycobacteria Unit, Institut Pasteur de Guadeloupe, Morne Joliviere, BP 484, 97183-Abymes, Cedex, Guadeloupe, France; 8 Laboratorio de Salud Pública, Dirección General de Salud Pública, Ramón y Cajal n° 68, 50004 Zaragoza, Spain

## Abstract

**Background:**

A large and unsuspected tuberculosis outbreak involving 18.7% of the total of the tuberculosis cases studied, was detected in a population-based molecular epidemiological study performed in Zaragoza (Spain) from 2001 to 2004.

**Methods:**

The *Mycobacterium tuberculosis *drug-susceptible strain, named *MTZ *strain, was genetically characterized by IS*6110*-RFLP, Spoligotyping and by MIRU-VNTR typing and the genetic patterns obtained were compared with those included in international databases. The characteristics of the affected patients, in an attempt to understand why the *MTZ *strain was so highly transmitted among the population were also analyzed.

**Results:**

The genetic profile of the *MTZ *strain was rare and not widely distributed in our area or elsewhere. The patients affected did not show any notable risk factor for TB.

**Conclusion:**

The *M. tuberculosis *strain *MTZ*, might have particular transmissibility or virulence properties, and we believe that greater focus should be placed on stopping its widespread dissemination.

## Background

*Mycobacterium tuberculosis *is an extremely successful pathogen that kills nearly two million people in the world each year [1]. The study of tuberculosis (TB) epidemiology and transmission, traditionally involving patient contact tracing, has been improved by the use of molecular strain typing [2]. Molecular epidemiological studies have added much-needed accuracy and precision to the study of transmission dynamics, and have allowed previously unresolved issues to be newly addressed, e.g. the classification of recent-versus-reactive disease, the extent of exogenous reinfection and the detection of unsuspected transmission events [2].

Advances in molecular typing have led to the identification of highly transmissible *M. tuberculosis *complex strains in the last years. In New York City, the W strain caused more than 350 cases [3] and spread to other American states [4]. A Beijing strain imported from Liberia affected 75 patients in the Gran Canaria Island during the 1990s [5]. Currently, the major threat for TB control is the transmission of extensively drug-resistant (XDR) strains [6,7]. However other strains, neither belonging to the W-Beijing family nor being drug-resistant, have shown extensive dissemination in various countries: the C strain in New York City [8,9], the CH strain in the UK [10,11], the Harlingen strain in the Netherlands [12,13] and the Danish Cluster 1 and 2 strains in Denmark [14].

In Zaragoza (Spain), an unsuspected, extensive transmission of a drug-susceptible *M. tuberculosis *strain was detected during a population-based molecular epidemiological survey from 2001 to 2004 [15]. In this study, 454 *M. tuberculosis *isolates were analyzed by IS*6110*-RFLP, and 52.6% were clustered. The largest cluster contained 85 isolates (18.7%); the strain causing this cluster was named *MTZ, Mycobacterium tuberculosis Zaragoza*.

The reasons for the dominance and widespread of the *MTZ *strain were unclear. The objectives of the present work were: i) to characterize the *MTZ *isolates by other genotyping techniques such as Spoligotyping and MIRU-VNTR typing, and include it in a Principal Genotype Group (PGG) ii) to compare the genetic patterns obtained with those included in international databases and iii) to analyze the characteristics of the affected patients, in an attempt to understand why the *MTZ *strain was so highly transmitted among the population.

## Methods

The *MTZ *cluster was identified in a population-based study conducted in the province of Zaragoza between 1^st ^June 2001 and 31^st ^May 2004. A total of 454 *M. tuberculosis *complex isolates were typed by IS*6110*-RFLP: 239 isolates were grouped into 45 clusters, each cluster containing between two and 85 isolates [15].

### DNA fingerprinting

#### *IS6110*-RFLP

RFLP analysis of the 85 isolates by Southern blotting and DNA hybridization with IS*6110 *was performed in the previous study according to the standard fingerprinting method [16]. The RFLP pattern was entered into the Spanish Database of the University of Zaragoza. This database includes 5,694 IS*6110*-RFLP entries from drug-susceptible and drug-resistant *M. tuberculosis *complex isolates, 4,637 (81%) of which are from Spanish isolates. The *MTZ *RFLP pattern was compared with the other database entries.

#### Spoligotyping

Spoligotyping was performed according to the method described by Kamerbeek *et al. *[17]. The spoligotype obtained was contrasted with entries contained in the SpolDB4 database [18] and was further compared with the updated SITVIT2 database. SITVIT2 is a proprietary database maintained at the Pasteur Institute of Guadeloupe, which contains both spoligotype and MIRU-VNTR patterns of the *M. tuberculosis*. At the time of this comparison (30th October 2008), it contained data on about 70,000 strains from 160 countries of origin.

#### MIRU-VNTR

The 85 isolates were genotyped by PCR amplification of a highly discriminatory subset of 15 loci proposed by Supply *et al. *[19]. Analyses were performed using five multiplex PCRs (Table 1), PCR mixtures and conditions were as described in reference 19 with some modifications. PCR products were separated on a 48-capillary MegaBACE™ 500 Sequencer (GE Healthcare Life Sciences) using Rox-labeled MegaBACE ET900-R as a size standard. PCR fragments sizes were determined using the MegaBACE™ Fragment Profiler v1.2 software (GE Healthcare Life Sciences). VNTR alleles were assigned according to size offsets, which correct the differences in relative migration between the size standard and the amplicons. Agarose gel electrophoresis with PCR products of known size were used to define size offsets.

**Table 1:** PCR mixtures and conditions used for the MIRU-VNTR genotyping

**Multiplex**	**Locus**	**Alias**	**VNTR length (bp)**	**[MgCl_2_] (mM)**	**PCR primer pairs (5' to 3'), with labelling indicated in brackets^a^**
Mix 1	580	MIRU 4	77	3	GCGCGAGAGCCCGAACTGC (FAM) GCGCAGCAGAAACGCCAGC
	2996	MIRU 26	51	3	TAGGTCTACCGTCGAAATCTGTGAC CATAGGCGACCAGGCGAATAG (HEX)
	802	MIRU 40	54	3	GGGTTGCTGGATGACAACGTGT (TAMRA) GGGTGATCTCGGCGAAATCAGATA

Mix 2	960	MIRU 10	53	2	GTTCTTGACCAACTGCAGTCGTCC GCCACCTTGGTGATCAGCTACCT (FAM)
	1644	MIRU 16	53	2	TCGGTGATCGGGTCCAGTCCAAGTA CCCGTCGTGCAGCCCTGGTAC (HEX)
	3192	MIRU 31	53	2	ACTGATTGGCTTCATACGGCTTTA GTGCCGACGTGGTCTTGAT (TAMRA)

Mix 3	424	Mtub04	51	1.5	CTTGGCCGGCATCAAGCGCATTATT GGCAGCAGAGCCCGGGATTCTTC (FAM)
	577	ETR C	58	1.5	CGAGAGTGGCAGTGGCGGTTATCT (HEX) AATGACTTGAACGCGCAAATTGTGA
	2165	ETR A	75	1.5	AAATCGGTCCCATCACCTTCTTAT (TAMRA) CGAAGCCTGGGGTGCCCGCGATTT

Mix 4	2401	Mtub30	58	3	CTTGAAGCCCCGGTCTCATCTGT (FAM) ACTTGAACCCCCACGCCCATTAGTA
	3690	Mtub39	58	3	CGGTGGAGGCGATGAACGTCTTC (HEX) TAGAGCGGCACGGGGGAAAGCTTAG
	4156	QUB-4156	59	3	TGACCACGGATTGCTCTAGT GCCGGCGTCCATGTT (TAMRA)

Mix 5	2163b	QUB-11b	69	1.5	CGTAAGGGGGATGCGGGAAATAGG CGAAGTGAATGGTGGCAT (FAM)
	1955	Mtub21	57	1.5	AGATCCCAGTTGTCGTCGTC (HEX) CAACATCGCCTGGTTCTGTA
	4052	QUB-26	111	1.5	AACGCTCAGCTGTCGGAT (TAMRA) CGGCCGTGCCGGCCAGGTCCTTCCCGAT

^a^ VIC and NED labeling used in reference 19 have been replaced by HEX (5'-hexachloro-fluorescein phosphoramidite) and TAMRA (6'-carboxy-tetramethyl-rhodamine), respectively.

Two *MTZ *isolates were also genotyped by PCR amplification of the 12 "old" loci initially described [20], six of which were also present in the 15 MIRU-VNTR panel and compared with the updated SITVIT2 database.

The 21 loci analysed were sent to be compared to a web server, MIRU-VNTRplus [21], that includes a collection of 186 strains representing the major MTBC lineages. For 15 different MIRU-VNTR it was also possible the comparison with the patterns included in MLVA database .

### Assignation of the *MTZ *strain to one of the three principal genotypic groups

*M. tuberculosis MTZ *strain was assigned to one of the three PGG delineated by Sreevatsan *et al*. [22]. Polymorphism at codon 463 of the *katG *gene was evaluated by PCR amplification of a 620 bp portion of the gene with the forward primer katG904 (5'-AGCTCGTATGGCACCGGAAC) and the reverse primer katG1523 (5'-TTGACCTCCCACCCGACTTG) [23], followed by digestion with *MspI*. In the presence of the CGG variant of codon 463, a *MspI *recognition site is formed, and the two alleles are easily differentiated by their restriction patterns.

Polymorphism at codon 95 of the *gyrA *gene was detected by PCR amplification of a 320 bp fragment using the primers gyrA1 (5'-CAGCTACATCGACTATGCGA) and gyrA2 (5'-GGGCTTCGGTGTACCTCAT) [24] followed by DNA sequencing.

### Information and statistical analysis of the patients

Medical and laboratory records of the patients were retrospectively and thoroughly reviewed. The primary routine contact investigation reports of the TB surveillance system in Zaragoza were also collected. The information included demographic data (age, sex, country of origin, place of residence), microbiological data (date of isolation and drug sensitivity), clinical data (site of disease), risk factors for TB (homelessness, injection drug use, alcohol and/or tobacco abuse, presence of HIV infection and history of previous imprisonment), and other information about possible epidemiological links.

The characteristics of the 85 patients in the *MTZ *cluster were compared with the other 369 TB patients studied in 2001–2004 [15]. We used the chi-square test (Yates-corrected) or Fisher's exact test to compare categorical data. The Mann-Whitney non-parametric test was used to compare the distribution of age as a continuous variable. The SPSS program for Windows (version 11.5; SPSS Inc, Chicago Il) was used for statistical analyses.

## Results and discussion

### DNA fingerprinting

**Figure 1:**
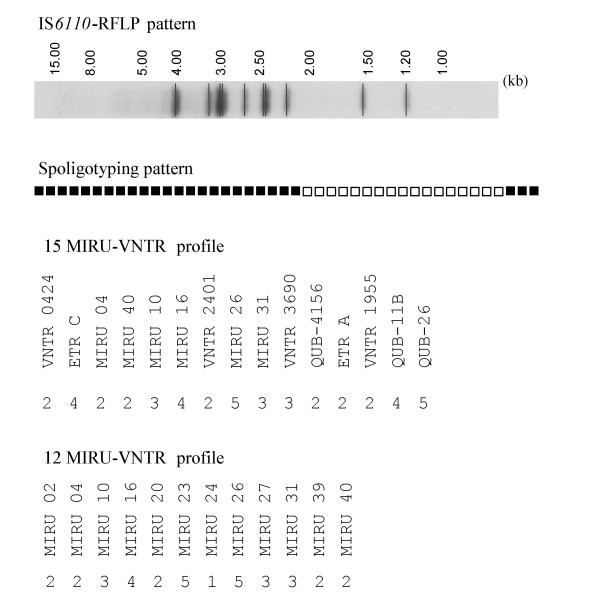
**IS*6110*-RFLP pattern, Spoligotyping pattern and MIRU-VNTR profiles of the *MTZ *strain**.

The 85 isolates of the *MTZ *cluster all showed the same IS*6110*-RFLP pattern, Spoligotype pattern and MIRU-VNTR pattern. The genetic profiles are shown in Figure 1. RFLP detected ten copies of IS*6110 *in the *MTZ *strain; this pattern was previously identified in only two patients from Zaragoza in 1993 and 1995 [25,26] and did not match any other profiles included in the database of the University of Zaragoza. Thus it is likely that this strain spread recently and was not endemic in our region during the nineties.

*M. tuberculosis MTZ *strain was assigned to one of the three PGG (22) trying to provide a better framework for phylogenetic of this *M. tuberculosis *strain. The SNPs in codon *KatG*463 (CGG) and codon *gyrA*95 (AGC) revealed that the *MTZ *strain belonged to the principal genetic group 3.

The *MTZ *spoligotyping profile did not belong to any of the predominant spoligotype families from Spain, Europe or any other region of the world and did not match highly transmissible profiles like the W-Beijing [18]. The spoligotyping profile corresponded to the SIT number 773 in the SpolDB4 database (octal number 777777760000031), with no related genetic family identified and only five isolates reported (four from New York City, USA, and one from Jakarta, Indonesia) [18]. Further comparison with the updated database did not show any new additions since the last 5 strains described in SpolDB4. The search for 12-loci MIRU pattern 223425153322 showed that it belonged to a widely reported shared-type MIT157 in the updated SITVIT2 database; it was shared by a total of 61 strains from 9 different settings: Croatia (20/61 or 33%), Belgium (16/61 or 26%), Great Britain (11/61 or 18%), Spain (3/61 or 5%), French overseas departments of the Americas (Guadeloupe, Martinique, French Guiana; 5/61 or 8%), Sweden (2/61 or 3%), South Africa (2/61 or 3%), USA (1/61 or 1.5%), and Italy (1/61 or 1.5%). Spoligotypes were available for only 31/61 strains sharing MIT157, with the following distribution of the genotypic lineages: T lineage (25/31 or 80%), Haarlem (4/31 or 13%), LAM (1/31 or 3%), and Beijing (1/31 or 3%). The signature of the SIT773 contained a very unusual absence of spacers 37 to 40, which does not denote any known lineage-specific signature in SpolDB4 or SITVIT2 databases. The overall pattern of SIT773 has a resemblance to the "Zero copy" lineage described previously (18), nonetheless, the fact that MIRU24 value is equal to "1" together with the assignation at the group 3 of the PGG, suggest that the *MTZ *strain might be an evolutionary "modern" strain of tubercle bacilli. Further investigations using SNPs and 24-loci MIRU-VNTR would be necessary to determine the exact phylogeographical specificity of the strains containing this SIT773 pattern.

The comparison to MIRU-VNTRplus web server  (21), and with MLVA database in , did not report any new information.

The molecular typing of the 85 isolates by Spoligotyping and MIRU-VNTR typing was also useful to confirm the composition of the large *MTZ *cluster initially identified by IS*6110*-RFLP.*M. tuberculosis *isolates from epidemiologically linked patients generally show identical IS*6110*-RFLP patterns; however, IS*6110*-based RFLP fingerprints are not always reliable indicators of epidemiological linkage [27-29], and MIRU-VNTR typing can subdivide IS*6110*-RFLP clusters with no epidemiological connections [19,30]. The epidemiological information on the *MTZ *cases was limited, because the molecular typing was retrospective and subsequent to the contact tracing and the patients were not re-interviewed. Despite the absence of epidemiological links (only small groups of patients, constituting a total of 26 patients altogether, were clearly linked) (Table 2), the MIRU typing did not separate the cluster of 85 isolates, suggesting that all the patients studied could be connected.

**Table 2:** Characteristics of epidemiologically linked patients of the *MTZ *cluster

**ID n°**	**Age (years)**	**Sex**	**Date of isolation**	**HIV status**	**Origin**	**Epidemiological link**	**Area of residence^a^**
121	43	Man	200402	Negative	Spain	Family contact	10
130	38	Woman	200402	Negative	Spain	Children attending the same nursery	10
							
140	26	Woman	200404	Negative	Spain	Nursery staff ^b^	-
449	1	Man	200404	Unknown	Spain	Children attending the same nursery	1
443	2	Woman	200404	Unknown	Spain	Children attending the same nursery	3
437	1	Man	200404	Unknown	Spain	Children attending the same nursery	1
445	1	Man	200404	Unknown	Spain	Children attending the same nursery	2
148	2	Woman	200404	Unknown	Spain	Children attending the same nursery	1
441	2	Woman	200404	Unknown	Spain	Children attending the same nursery	2
444	1	Man	200404	Unknown	Spain	Children attending the same nursery	1
442	1	Woman	200404	Unknown	Spain	Children attending the same nursery	4
							
180	2	Woman	200108	Unknown	Spain	Family contact	-
186	20	Woman	200109	Negative	Spain		18b
159	35	Man	200106	Negative	Spain		-
							
269	26	Woman	200207	Negative	Algeria	Family contact	16
257	43	Man	200206	Negative	Spain		16
							
272	51	Man	200208	Unknown	Spain	Family contact	18c
285	19	Man	200209	Negative	Spain		18c
							
160	41	Man	200106	Negative	Spain	Family contact	18d
341	5	Man	200304	Unknown	Spain		18d
							
201	27	Man	200111	Negative	Spain	Family contact	17
196	23	Woman	200110	Negative	Spain		17
							
422	24	Woman	200402	Unknown	Spain	Family contact	7
419	8 months	Woman	200402	Negative	Spain		7
							
36	25	Man	200205	Positive	Spain	Friendship contact	-
56	25	Man	200209	Unknown	Spain		-

^a ^The areas of residence are shown in figure 2. The dash (-) means that the patient did not live in any of the 18 areas of residence (see figure 2). ^b ^The parents of the woman who worked at the nursery lived close to area n° 5. The woman also worked for 7 years in a bingo hall in area 18d, and for one year in a bingo hall between the areas 7 and 8.

n°: patient identification number.

### Characteristics and epidemiological investigation of the patients

Sixty (70.6%) of the 85 cases of the *MTZ *cluster were men and 25 (29.4%) were women. The median age of the patients was 30 years (25^th ^percentile [*P*_25_] to *P*_75_, 23 to 41) with a range of 8 months to 68 years. Seventy-three (86%) patients were born in Spain; 12 (14%) were foreign-born. All patients presented with a drug-susceptible isolate, except one patient with history of relapse and non-adherence to the TB treatment, who presented a rifampicin-resistant isolate.

We compared the characteristics of the *MTZ*-infected patients with the rest of clustered patients from the previous study (154 cases) and with total patients studied (clustered and non-clustered, 369 cases). The median age of *MTZ*-infected patients was lower (30 years) than that of other clustered patients (median age 38 years; *P*_25 _to *P*_75_, 29 to 46) or than that of all patients (median age 40 years; *P*_25 _to *P*_75_, 30 to 62) (*P *< 0.001 in both comparisons). Almost all (94.1%) of the *MTZ*-infected patients lived in Zaragoza city; the corresponding values were 81.5% for other clustered patients (*P *= 0.012) and 76.1% for all patients (*P *< 0.001). The *MTZ*-infected patients were less likely to be injection drug users (9.9%) than other clustered patients (22.9%) (*P *= 0.024), and presented more frequently with pulmonary TB than all other patients (95.3% *vs. *85.1%; *P *= 0.019). In many TB outbreaks, the patients share a common risk factor for TB such as homelessness, HIV infection, injection drug abuse or alcohol abuse [5-9,31]. However, in our study, *MTZ *cluster patients do not appear to be linked by any common characteristic or risk factors for TB. Other European studies have reported the dissemination of TB strains among low-risk individuals, such as the Harlingen strain in the Netherlands or the Danish cluster 2 strain in Denmark [12-14].

A number of epidemiological links could be established from the data available from patient medical records and contact investigation reports. Eight children (all between one and two years old) and one nursery staff were linked because they attended the same nursery [32]. Familial links were identified involving 15 household contacts in seven different families and a friendship link between two other patients. Patient characteristics are detailed in Table 2.

**Figure 2:**
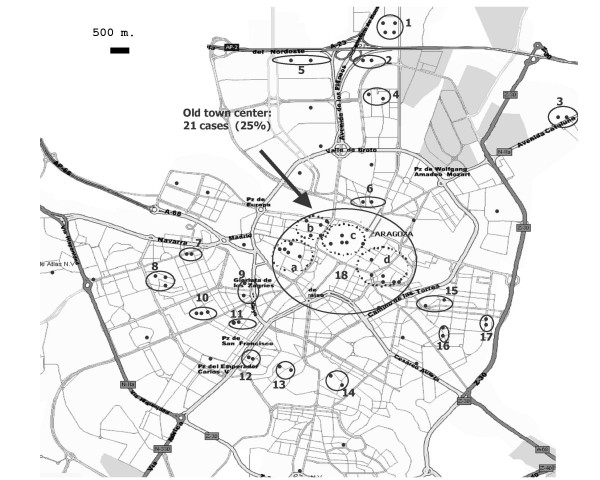
**Location of 78 cases of the *MTZ *cluster in Zaragoza City according to the place of residence**. The cases grouped inside a circle lived in the same street, or in parallel, adjoining or perpendicular streets. The areas that included two or more cases are numbered from 1 to 18.

A significant data was the proportion of patients linked by place of residence. The study area included the province of Zaragoza [15], which is composed of both urban and rural areas, but 94.1% of the *MTZ*-infected patients lived in Zaragoza city. Thirty-nine patients were linked into groups of two or three individuals because they lived in the same street, or in parallel, adjoining or perpendicular streets (Table 3). Seventy-eight cases of the *MTZ *cluster were situated in the city of Zaragoza, and 21 (25%) of these were concentrated in the old town centre (Figure 2). The other seven patients lived in peripheral districts or in rural areas. Other studies have demonstrated that patients whose residences are geographically aggregated may be clustered [33] and therefore, the location of TB exposure is an important factor to consider in addition to the contact tracing [9,34,35]. TB transmission between sporadic or casual contacts has been documented [29,31]; hypothetically, the transmission of the *MTZ *strain could have occurred casually in public settings, probably near to the residences of patients.

**Table 3:** Characteristics of the *MTZ *cluster patients linked by the place of residence and/or by common risk factors for TB

**ID n°**	**Age (years)**	**Sex**	**Date of isolation**	**HIV status**	**Origin**	**Area of Residence ^a^**	**Characteristics in common ^b^**
371	30	Man	200307	Negative	Guinea	11	Neighbours
							
42	25	Man	200206	Negative	Spain	11	
177	32	Woman	200109	Negative	Spain	18d	Neighbours
							
171	38	Man	200108	Negative	Spain	18d	
60	44	Man	200209	Negative	Spain	-	Prison, alcoholism
258	36	Man	200205	Positive	Spain	-	Prison, alcoholism
167	47	Man	200107	Unknown	Spain	-	Prison
246	33	Man	200204	Negative	Spain	18b	Neighbour of 322
322	27	Man	200303	Negative	Egypt	18b	Prison, neighbour of 246, IDU
69	24	Man	200211	Negative	Unknown	18a	Neighbours, alcoholism
263	53	Man	200207	Negative	Spain	18a	Neighbours, alcoholism
							
374	46	Man	200308	Positive	Spain	18a	Ex-IDU
217	34	Man	200112	Positive	Unknown	-	Ex-IDU
23	36	Man	200112	Positive	Spain	-	Ex-IDU
304	34	Man	200303	Negative	South America	-	Ex-IDU
							
79	26	Man	200212	Positive	Venezuela	6	-
434	56	Man	200403	Unknown	Spain	6	Alcoholism. Worked next to area 5
271	39	Man	200208	Negative	Spain	-	Alcoholism
178	47	Man	200109	Negative	Spain	3	Alcoholism
							
428	26	Woman	200402	Positive	Spain	4	Lived in the same area than 442 (table 2)
							
318	44	Man	200303	Negative	Gambia	-	15 years in Spain
312	25	Man	200302	Negative	Gambia	10	10 years in Spain. Worked in area 8.
							
59	39	Woman	200209	Negative	Spain	8	
76	43	Man	200212	Unknown	Ukraine	8	
125	60	Woman	200402	Negative	Spain	8	
							
302	41	Man	200301	Negative	Spain	15	
427	46	Man	200402	Negative	Spain	15	
							
94	50	Man	200307	Negative	Spain	9	
194	57	Man	200109	Negative	Spain	9	
							
209	31	Man	200112	Negative	Spain	12	
283	26	Man	200209	Negative	Spain	12	
							
174	36	Woman	200108	Negative	Spain	13	
205	26	Woman	200111	Negative	Spain	13	
							
342	21	Man	200305	Negative	Spain	14	
276	35	Man	200209	Negative	Spain	14	
							
237	49	Man	200204	Positive	Spain	5	
297	28	Man	200210	Negative	Unknown	5	
							
247	36	Woman	200205	Positive	Spain	18a	
199	63	Man	200111	Unknown	Spain	18a	
385	44	Man	200309	Positive	Spain	18b	
18	68	Man	200111	Unknown	Spain	18b	
431	29	Woman	200402	Positive	Spain	18c	
214	53	Man	200201	Negative	Spain	18c	
370	52	Man	200308	Negative	Spain	18c	
26	31	Woman	200201	Negative	Spain	18c	
260	33	Man	200206	Negative	Spain	18d	
158	20	Woman	200106	Unknown	Spain	18d	

^a ^The areas of residence are shown in figure 2. The dash (-) means that the patients did not live in any of the 18 areas of residence (see figure 2). ^b ^Patients were classified as "neighbours" when they lived in the same street.

ID n°: patient identification number. IDU: injection drug user.

In one of the small outbreaks caused by *MTZ *strain registered among children attending at a nursery, 11.7% showed a positive skin test and 90.9% of them developed the illness [32]. However, the percentage of infected children was surprisingly low in view of the dissemination of this strain among the total population in Zaragoza. Like strains involved in other outbreaks, the *MTZ *strain might have unique characteristics for virulence and/or transmissibility [36-39]. The transmission of tuberculosis may have been due to the increased virulence of the strain rather than to environmental factors or patient characteristics.

Another possible explanation for the extensive spread of the *MTZ *strain could be that this strain was endemic in our region, but in a previous molecular study performed in the same area between 1993 and 1995 [25,26], *MTZ *was only isolated from two Spanish patients who were not apparently connected; one in 1993 and one in 1995. The first patient was an 11 year-old girl living in a rural area, and the second was a 22 year-old woman living in the old town centre in Zaragoza. Surprisingly, both patients presented with rare extra-pulmonary locations of TB (bone TB and intestinal TB respectively), whereas 95.3% of the cases reported from 2001 to 2004 presented with pulmonary TB. The period from 1995 to 2001 has not been studied, and it seems likely that more *MTZ *cases would be identified during this period. A more detailed epidemiological investigation of the outbreak is needed to elucidate the chain of transmission.

## Conclusion

In conclusion, neither the genetic profiles exhibited by the *MTZ *strain nor the characteristics of the patients affected could explain the reasons for the dominance and widespread of this modern strain. The *MTZ *strain might have particular transmissibility or virulence properties that need to be studied, and we believe that greater focus should be placed on stopping its widespread dissemination. Molecular typing has been a decisive tool to detect this unsuspected TB outbreak, and demonstrates the importance of the combination of both traditional approaches and molecular epidemiology for TB surveillance.

## Competing interests

The authors declare that they have no competing interests.

## Authors' contributions

SS and AILC designed the study, supervised all the experimental work, analyzed the results, wrote the manuscript. AILC and PG performed the experimental assays. MALe and MAV compiled all the isolates and the first clinical and epidemiological data. JG, MALa collect all the clinical and epidemiological data. MJI helped in analysis of results. NR helped in the global analysis of the genotype. CM and MJR critically reviewed the final version of the manuscript. All authors read and approved the final manuscript.

## Pre-publication history

The pre-publication history for this paper can be accessed here:


